# Higher HbA_1c_ variability is associated with increased arterial stiffness in individuals with type 1 diabetes

**DOI:** 10.1186/s12933-023-01770-2

**Published:** 2023-03-04

**Authors:** Anniina Tynjälä, Valma Harjutsalo, Fanny Jansson Sigfrids, Per-Henrik Groop, Daniel Gordin, the FinnDiane Study Group

**Affiliations:** 1 Folkhälsan Institute of Genetics, Folkhälsan Research Center, Helsinki, Finland; 2 Department of Nephrology, University of Helsinki and Helsinki University Hospital, Helsinki, Finland; 3 Research Program for Clinical and Molecular Metabolism, Faculty of Medicine, University of Helsinki, Helsinki, Finland; 4 Minerva Foundation Institute for Medical Research, Helsinki, Finland; 5 Department of Diabetes, Central Clinical School, Monash University, Melbourne, Australia; 6 Joslin Diabetes Center, Harvard Medical School, Boston, MA USA

**Keywords:** Arterial stiffness, Applanation tonometry, Augmentation index, Cardiovascular disease, Glycaemic variability, HbA_1c_, Pulse wave velocity, Type 1 diabetes

## Abstract

**Background:**

Both long-term glycaemic variability and arterial stiffness have been recognized as cardiovascular risk factors. This study aims to investigate whether an association between these phenomena exists in individuals with type 1 diabetes.

**Methods:**

This cross-sectional study included 673 adults (305 men, 368 women) with type 1 diabetes and combined available retrospective laboratory data on HbA_1c_ from the preceding 10 years with outcome data on arterial stiffness and clinical variables from a comprehensive study visit. HbA_1c_ variability was calculated as adjusted standard deviation (adj-HbA_1c_-SD), coefficient of variation (HbA_1c_-CV) and average real variability (HbA_1c_-ARV). As measures of arterial stiffness, carotid-femoral pulse wave velocity (cfPWV; n = 335) and augmentation index (AIx; n = 653) were assessed using applanation tonometry.

**Results:**

The study population had a mean age of 47.1 (± 12.0) years and a median duration of diabetes of 31.2 (21.2–41.3) years. The median number of HbA_1c_ assessments per individual was 17 (12–26). All three indices of HbA_1c_ variability were significantly correlated with both cfPWV and AIx after adjustment for sex and age (*p* < 0.001). In separate multivariable linear regression models, adj-HbA_1c_-SD and HbA_1c_-CV were significantly associated with cfPWV (*p* = 0.032 and *p* = 0.046, respectively) and AIx (*p* = 0.028 and *p* = 0.049, respectively), even after adjustment for HbA_1c_-mean. HbA_1c_-ARV was not associated with cfPWV or AIx in the fully adjusted models.

**Conclusions:**

An association independent of HbA_1c_-mean was found between HbA_1c_ variability and arterial stiffness, suggesting a need to consider multiple HbA_1c_ metrics in studies assessing cardiovascular risk in type 1 diabetes. Longitudinal and interventional studies are needed to confirm any causal relationship and to find strategies for reducing long-term glycaemic variability.

## Background

Despite marked reduction in cardiovascular morbidity and mortality during the last decades, type 1 diabetes is still associated with a significant burden of cardiovascular disease (CVD) compared to the general population [1]. CVD remains the leading cause of death and reduced life-expectancy in individuals with type 1 diabetes [2], and the excess cardiovascular mortality is evident even in the absence of kidney disease [3]. Considering differences in pathophysiology and the cardiovascular risk profile compared to type 2 diabetes, it is important to assess the cardiovascular risk factors in individuals with type 1 diabetes separately. Clinical markers beyond the established cardiovascular risk factors are needed for early recognition and prevention of the excess cardiovascular morbidity.

The importance of intensive glycaemic control for the prevention of CVD was shown by the Diabetes Control and Complications Trial/Epidemiology of Diabetes Interventions and Complications (DCCT/EDIC) Study [4]. However, Swedish registry data imply that individuals with type 1 diabetes and an on-target mean HbA_1c_ (≤ 52 mmol/mol) still have a risk of cardiovascular death twice as high compared to the general population [5]. On the other hand, a clinical trial in individuals with type 2 diabetes, the Action to Control Cardiovascular Risk in Diabetes (ACCORD) trial, was discontinued after an increase in the mortality in those individuals receiving intensive treatment of hyperglycaemia [6]. A recent post-hoc analysis of ACCORD revealed, interestingly, long-term glycaemic variability (GV) to be an independent risk factor for cardiovascular outcomes [7]. The view of hyperglycaemia as a risk factor has broadened beyond single or mean values of blood glucose and HbA_1c_, and research efforts have paid special attention to both short-term GV, typically measured by continuous glucose monitoring (CGM; [8]), and long-term GV, most commonly assessed as HbA_1c_ variability. Indeed, HbA_1c_ variability has been associated with the development of CVD and/or increased mortality in type 1 diabetes [9–12]. An interesting question is, whether lower cardiovascular mortality observed in association with continuous subcutaneous insulin infusion (CSII) therapy in the Swedish registry data could be attributed to lower glycaemic variability [13].

The mechanisms by which long-term GV adds to the risk of vascular complications are not completely understood. Some of the proposed mediating factors, such as oxidative stress and endothelial dysfunction [14], also play a central role in arterial stiffness [15], which is a surrogate marker of CVD recently recognized also in type 1 diabetes [16–18]. Previous studies suggest arterial stiffness to be associated with short-term GV in type 2 diabetes and the general population [19, 20], as well as with long-term variability of fasting plasma glucose in the general population [21]. In individuals with type 1 diabetes, three studies with small sample sizes did not detect any statistically significant association between CGM-derived short-term GV metrics and arterial stiffness [22–24]. Only one recent study with 54 individuals has investigated long-term GV in relation to arterial stiffness, and did not find any association between HbA_1c_ variability and arterial stiffness in type 1 diabetes [24]. Larger studies are needed to make conclusions about the association between GV and arterial stiffness in type 1 diabetes.

## Methods

### Aim and design

The aim of this study is to find out whether there is an association between HbA_1c_ variability and arterial stiffness in individuals with type 1 diabetes, where we hypothesize that a highly variable HbA_1c_ is associated with increased arterial stiffness. The study design is cross-sectional with a study visit including clinical characterization, assessment of the outcome variables (arterial stiffness) and a retrospective view on the exposure variables (HbA_1c_ trajectories).

### Study population

FinnDiane (The Finnish Diabetic Nephropathy Study) is an ongoing prospective multi-centre cohort study with the aim of identifying risk factors associated with the chronic complications of type 1 diabetes. The study protocol was approved by the Helsinki and Uusimaa Hospital District Ethics Committee and written informed consent is obtained from each participant. To date, more than 5400 individuals with type 1 diabetes have been characterized within the cohort. For the subset of participants studied in Helsinki, assessment of arterial stiffness is included in the protocol. Data from the participants are collected during recurring study visits including comprehensive clinical and laboratory measurements, as well as standardized questionnaires on medication and diabetic complications. In this study, history of retinal laser treatment was used as a proxy for retinopathy. Kidney failure was defined as kidney replacement therapy or eGFR < 15 ml/min/1.73 m^2^ estimated by the Chronic Kidney Disease Epidemiology Collaboration (CKD-EPI) formula. CVD events were defined as myocardial infarction, coronary revascularization, stroke, lower extremity revascularization or non-traumatic amputation.

The main inclusion criteria of this study were age over 18 years, the onset of diabetes before the age of 40, and initiation of insulin-treatment within one year from diagnosis. Considering the scope of this study, further inclusion criteria were a minimum of five HbA_1c_ measurements available from a maximum period of 10 years prior to the assessment of arterial stiffness as part of a FinnDiane study visit in 2002–2019.

### Arterial stiffness

Carotid-femoral pulse wave velocity (cfPWV) is the gold standard measure for stiffness in the large arteries, whereas augmentation index (AIx) is a surrogate marker for stiffness in peripheral resistance arteries based on pulse wave reflection from the peripheral arterial tree [25]. In the FinnDiane participants studied in Helsinki, arterial stiffness is assessed using non-invasive applanation tonometry ([22]; SphygmoCor, Atcor Medical, Sydney, NSW, Australia). Pulse wave analysis, including the determination of AIx, has been part of the protocol from 2002, whereas the assessment of cfPWV was initiated later, and is available for those individuals studied from 2009 onwards.

For the assessment of cfPWV, a high-fidelity micromanometer (SPC-301; Millar Instruments, Houston, TX, USA) is used to consecutively record pulses at the carotid and femoral arteries with a simultaneous electrocardiogram as reference. The software takes the manually measured distances of both recording sites from the sternal notch as input (subtraction method) and calculates the cfPWV as the ratio of the estimated arterial path length and pulse transit time. AIx is determined by pulse wave analysis, where the peripheral pressure wave form is recorded at the radial artery and the central pressure wave form is generated by the software using a standardized transfer function. AIx is calculated from the central pressure wave form as the quotient of the augmentation pressure and the pulse pressure, the former representing the difference between the second and the first systolic peak, and expressed as a percentage. The values of AIx used in the analyses are adjusted to a heart rate of 75 beats per minute. The average of two valid measurements of cfPWV and three of AIx is used**.**

### HbA_1c_ variability

HbA_1c_ measurement by standardized assays is included in the laboratory assessment performed at each FinnDiane visit and additional available HbA_1c_ values are collected from the medical records. For this study, three indices of HbA_1c_ variability were calculated using retrospective laboratory data from the preceding 10 years up until the visit with the arterial stiffness assessment:

$${\text{adj-HbA}_{1\mathrm{c }}-SD}= \frac{ {\text{HbA}_{1\mathrm{c }}-SD}} { \sqrt{\frac{n}{n-1}} }$$

$${\text{HbA}_{1\mathrm{c }}-CV (\%)} =\frac{ {\text{HbA}_{1\mathrm{c }}-SD} }{ {\text{HbA}_{1\mathrm{c }}-mean} }\times 100\%$$

$${\text{HbA}_{1\mathrm{c }}-ARV}=\frac{1}{n-1}\times \sum_{k=1}^{n-1}\left|{\mathrm{HbA}}_{1\mathrm{c }\;k+1}-{\mathrm{HbA}}_{1\mathrm{c} \;k}\right|$$

To take into account the number of HbA_1c_ measurements (*n*), adjusted standard deviation (adj-HbA_1c_-SD) was used, and due to higher mean values being associated with higher standard deviation, also coefficient of variation (HbA_1c_-CV) was considered. HbA_1c_-CV is the standard deviation adjusted for the mean, expressed as a percentage. Average real variability (HbA_1c_-ARV) is the mean of the absolute differences between consecutive HbA_1c_ values [26] and gives information about visit-to-visit variability not captured by the other indices of variability. Each variability index was calculated both in the International Federation of Clinical Chemistry and Laboratory Medicine (IFCC) units (mmol/mol) and the DCCT-aligned National Glycohemoglobin Standardization Program (NGSP) units (%). For regression analyses, the IFCC units were used.

### Statistical methods

The data were analysed using IBM SPSS Statistics (version 27; IBM Corp., Armonk, NY, USA) and R open-source software (version 4.1.1, R Foundation for Statistical Computing, Vienna, Austria). Descriptive statistics for continuous variables are expressed as means ± SD for normally distributed, and as medians with IQR for non-normally distributed variables. For categorical variables, valid percentages are reported. Partial nonparametric correlations of potentially confounding clinical variables with arterial stiffness were calculated controlling for sex and age, and are reported as Spearman’s rank correlation coefficients (*r*_*s*_). Natural logarithmic transformations were used in the case of highly skewed variables in partial regression plots and linear regression analysis. The measures of arterial stiffness were regressed on each index of HbA_1c_ variability in separate multivariable linear regression models. After adjustment for sex and age, the stepwise variable selection method in SPSS was used to select further covariates into the model. The final model was additionally adjusted for HbA_1c_-mean. For missing data, pairwise deletion was used in correlation analyses and listwise deletion in regression analyses. In all analyses, *p* < 0.05 were considered statistically significant.

## Results

A total of 673 individuals (305 men, 368 women) were eligible and included in this study, with a mean age of 47.1 (± 12.0) years and a median duration of diabetes of 31.2 (21.2–41.3) years (Table 1). Of these individuals, 99 (14.9%) had experienced a CVD event, 76 (11.3%) had kidney failure and 37 (5.6%) individuals had both. The median number of HbA_1c_ assessments per individual was 17 [12–25, 27] from a retrospective follow-up time of 7.5 (5.5–9.1) years and with an HbA_1c_ assessment interval of 3.6 (2.0–5.6) months. The number of HbA_1c_ measurements was significantly associated with higher HbA_1c_-SD and HbA_1c_-CV but not with HbA_1c_-mean or HbA_1c_-ARV (data not shown). The median of intra-individual HbA_1c_-mean values was 65 mmol/mol (8.1%) and the median adj-HbA_1c_-SD was 6.5 mmol/mol (0.59%). Higher adj-HbA_1c_-SD, HbA_1c_-CV and HbA_1c_-ARV were all significantly correlated with higher HbA_1c_ at the endpoint visit as well as with higher mean-HbA_1c_ (data not shown), HbA_1c_-ARV having the highest correlation with HbA_1c_-mean (*r*_*s*_ = 0.538, *p* < 0.001). Of the outcome variables, 335 (49.7%) had cfPWV, and 653 (97.0%) had AIx available, with median values of 8.5 (7.1–10.8) m/s and 22 (13–28) %, respectively. Of those with cfPWV measurement available, 46.9% had a value exceeding 10 m/s, a cut-off value advised by an expert consensus [28]. CfPWV was missing for 338 participants mostly for obvious reasons described in the Methods section and AIx was missing or dropped for poor quality for 20 participants.

**Table 1:** Indices of HbA_1c_ variability, clinical characteristics and measures of arterial stiffness by subgroups

	All *n* = 673	cfPWV *n* = 335	Aix *n* = 653	Missing data
Retrospective follow-up of HbA_1c_				
HbA_1c_ assessments (*n*)	17 (12–26)	17 (12–28)	17 (12–26)	0
HbA_1c_-mean (mmol/mol)	65 (58–73)	63 (56–71)	64 (58–73)	0
HbA_1c_-mean (%)	8.1 (7.4–8.9)	7.9 (7.3–8.7)	8.1 (7.4–8.8)	0
Adj-HbA_1c_-SD (mmol/mol)	6.5 (4.5–8.9)	6.1 (4.3–8.3)	6.4 (4.5–8.8)	0
Adj-HbA_1c_-SD (%)	0.59 (0.41–0.81)	0.56 (0.40–0.76)	0.59 (0.41–0.80)	0
HbA_1c_-CV_IFCC_ (%)	10.2 (7.8–13.6)	9.6 (7.6–13.3)	10.1 (7.7–13.5)	0
HbA_1c_-CV_NGSP_ (%)	7.4 (5.6–10.0)	7.1 (5.3–9.7)	7.3 (5.5–10.0)	0
HbA_1c_-ARV (mmol/mol)	5.4 (4.1–7.2)	5.0 (3.8–6.7)	5.4 (4.1–7.1)	0
HbA_1c_-ARV (%)	0.50 (0.38–0.66)	0.45 (0.35–0.61)	0.49 (0.37–0.65)	0
Outcome visit				
Male sex (%)	305 (45.3)	163 (48.7)	296 (45.3)	0
Age (y)	47.1 ± 12.0	48.0 ± 11.6	47.1 ± 11.9	0
Diabetes duration (y)	31.2 (21.2–41.3)	33.3 (21.2–41.9)	31.2 (21.2–41.2)	0
Age at onset (y)	13.6 (9.7–20.9)	14.0 (9.7–21.1)	13.6 (9.7–20.9)	0
Height (cm)	171.5 ± 9.8	172.5 ± 10.0	171.5 ± 9.8	0
Waist-to-height ratio	0.51 (0.47–0.56)	0.51 (0.47–0.57)	0.51 (0.47–0.56)	11
BMI (kg/m^2^)	25.3 (23.0–28.2)	25.9 (23.5–28.7)	25.3 (23.0–28.1)	0
Systolic blood pressure (mmHg)	134 (123–148)	133 (120–145)	134 (123–148)	1
Diastolic blood pressure (mmHg)	75 (69–82)	74 (69–81)	75 (69–82)	1
Pulse pressure (mmHg)	57 (49–72)	55 (48–69)	57 (49–72)	1
Mean arterial pressure (mmHg)	95 (89–102)	94 (88–101)	95 (89–102)	1
Smoking (%)	77 (11.6)	25 (7.6)	76 (11.8)	12
HbA_1c_ (mmol/mol)	64 (56–73)	63 (56–71)	64 (57–73)	1
HbA_1c_ (%)	8.0 (7.3–8.8)	7.9 (7.3–8.6)	8.0 (7.4–8.8)	1
Total cholesterol (mmol/l)	4.5 (3.9–5.1)	4.4 (3.9–4.9)	4.5 (3.9–5.1)	0
HDL-cholesterol (mmol/l)	1.53 (1.24–1.87)	1.51 (1.22–1.86)	1.53 (1.24–1.87)	0
LDL-cholesterol (mmol/l)	2.4 (2.0–2.9)	2.3 (1.9–2.8)	2.4 (2.0–2.9)	0
Triglycerides (mmol/l)	0.94 (0.71–1.32)	0.92 (0.70–1.37)	0.94 (0.71–1.30)	0
eGFR (ml/min/1.73m^2^)	99 (78–111)	98 (79–111)	100 (79–111)	1
Nephropathy (%)	141 (21.5)	58 (18.2)	133 (20.8)	16
Kidney failure (%)	76 (11.3)	34 (10.1)	69 (10.6)	1
History of retinal laser treatment (%)	263 (39.3)	128 (38.2)	252 (38.8)	3
History of CVD events (%)	99 (14.9)	47 (14.2)	93 (14.4)	7
Antihypertensive therapy (%)	361 (53.8)	175 (52.6)	348 (53.5)	2
Statin therapy (%)	252 (37.7)	141 (42.5)	241 (37.1)	4
cfPWV (m/s)	8.5 (7.1–10.8)	8.5 (7.1–10.8)	8.6 (7.1–10.8)	338
AIx (%)	22 (13–28)	21 (13–27)	22 (13–28)	20

Data are means ± SD, medians (interquartile range) or proportions (valid percentage)

History of CVD (cardiovascular disease) events defined as myocardial infarction, coronary revascularization, stroke, lower extremity revascularization or non-traumatic amputation. Kidney failure defined as kidney replacement therapy or eGFR < 15 ml/min/1.73 m^2^. Nephropathy defined as severe albuminuria (urinary albumin excretion rate  ≥ 300 mg/24 h or ≥ 200 μg/min) or kidney failure

*cfPWV* carotid-femoral pulse wave velocity, *AIx* augmentation index, *adj-HbA*_*1c*_*-SD* adjusted standard deviation of HbA_1c_, *HbA*_*1c*_*-CV*_*IFCC*_ coefficient of variation of HbA_1c_ based on values in mmol/mol units, *HbA*_*1c*_*-CV*_*NGSP*_ coefficient of variation of HbA_1c_ based on values in % units, *HbA*_*1c*_*-ARV* average real variability of HbA_1c_

**Fig. 1:**
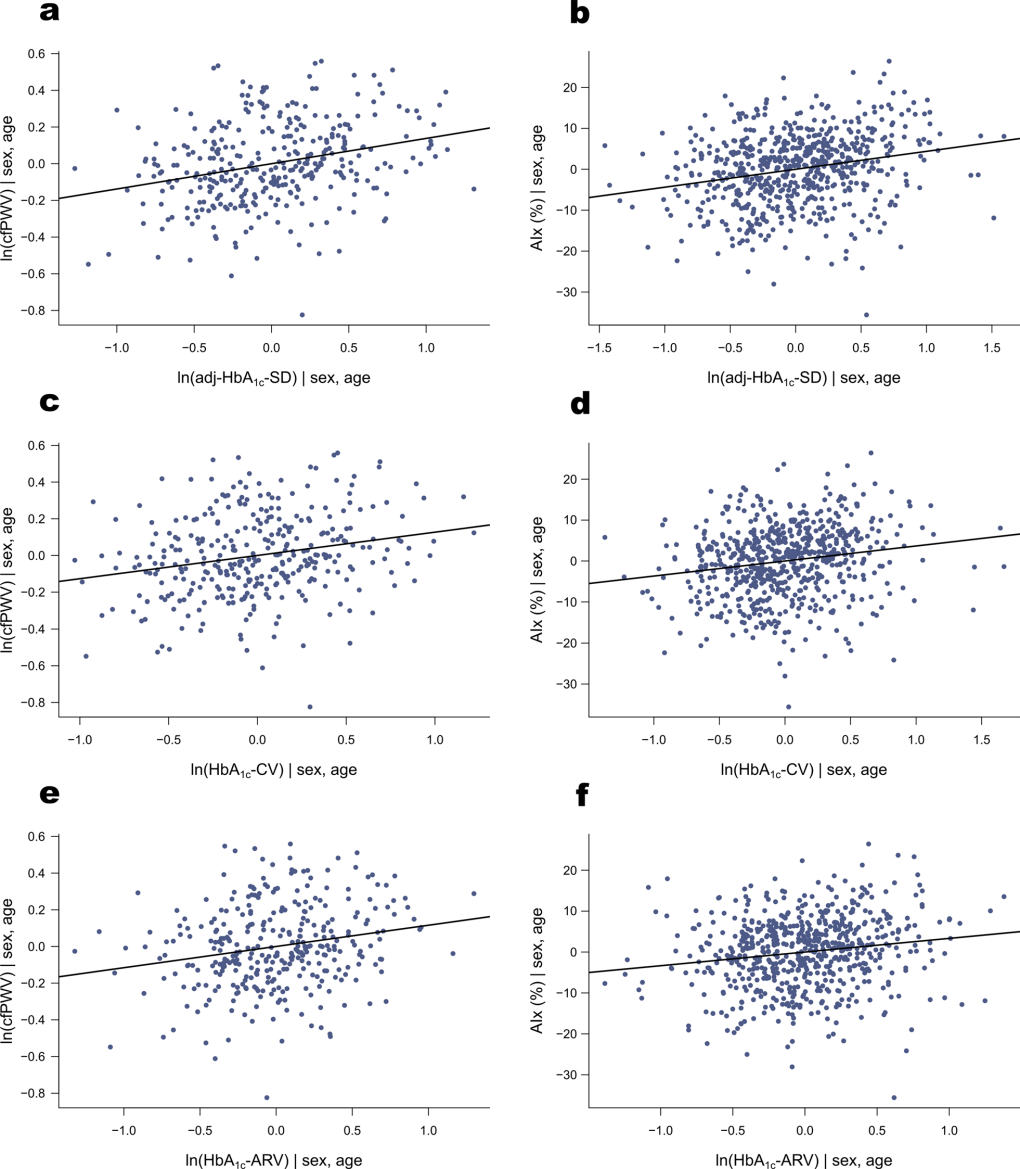
Sex- and age-adjusted partial regression plots of arterial stiffness and HbA_1c_ variability. *Adj-HbA*_*1c*_*-SD* adjusted standard deviation of HbA_1c_, *HbA*_*1c*_*-CV* coefficient of variation of HbA_1c_, *HbA*_*1c*_*-ARV* average real variability of HbA_1c_, *cfPWV* carotid-femoral pulse wave velocity, *AIx* augmentation index

In the sex- and age-adjusted nonparametric partial correlation analyses between each independent variable and the outcome variable, the variables with the highest correlations with cfPWV were age (*r*_*s*_ = 0.670), systolic blood pressure (SBP; *r*_*s*_ = 0.307) and eGFR (*r*_*s*_ = −0.288), whereas for AIx these were age (*r*_*s*_ = 0.585), sex (*r*_*s*_ = 0.431) and mean arterial pressure (MAP; *r*_*s*_ = 0.416). All three indices of HbA_1c_ variability were significantly (*p* < 0.001) correlated with both measures of arterial stiffness (Table 2). After natural log-transformations of cfPWV and the indices of HbA_1c_ variability, the associations were considered fairly linear by visual inspection of sex- and age-adjusted partial regression plots (Fig. 1).

**Table 2:** Sex- and age-adjusted partial Spearman correlations of HbA_1c_ variability and clinical characteristics with measures of arterial stiffness

	cfPWV (*n* = 335)		AIx (*n* = 653)	
	*r*_*s*_	*p* value	*r*_*s*_	*p* value
Retrospective follow-up of HbA_1c_				
HbA_1c_ assessments	0.144	0.008	0.177	0.003
HbA_1c_-mean (mmol/mol)	0.189	< 0.001	0.196	< 0.001
HbA_1c_-mean (%)	0.190	< 0.001	0.196	< 0.001
Adj-HbA_1c_-SD (mmol/mol)	0.278	< 0.001	0.245	< 0.001
Adj-HbA_1c_-SD (%)	0.278	< 0.001	0.246	< 0.001
HbA_1c_-CV_IFCC_ (%)	0.226	< 0.001	0.197	< 0.001
HbA_1c_-CV_NGSP_ (%)	0.242	< 0.001	0.212	< 0.001
HbA_1c_-ARV (mmol/mol)	0.197	< 0.001	0.173	< 0.001
HbA_1c_-ARV (%)	0.198	< 0.001	0.173	< 0.001
Outcome visit				
Sex (age-adjusted)	−0.116	0.034	0.431	< 0.001
Age (sex-adjusted)	0.670	< 0.001	0.585	< 0.001
Diabetes duration	0.257	< 0.001	0.110	0.005
Age at onset	−0.283	< 0.001	−0.101	0.010
Height	−0.040	0.467	−0.195	< 0.001
Waist-to-height ratio	0.145	0.008	0.106	0.007
BMI	0.100	0.070	0.002	0.957
Systolic blood pressure	0.307	< 0.001	0.384	< 0.001
Diastolic blood pressure	0.141	0.010	0.343	< 0.001
Pulse pressure	0.266	< 0.001	0.220	< 0.001
Mean arterial pressure	0.269	< 0.001	0.416	< 0.001
Smoking	0.041	0.462	0.130	< 0.001
HbA_1c_	0.171	0.002	0.144	< 0.001
Total cholesterol	−0.102	0.062	0.011	0.780
HDL-cholesterol	−0.032	0.564	−0.056	0.151
LDL-cholesterol	−0.151	0.006	−0.004	0.914
Triglycerides	0.118	0.031	0.155	< 0.001
eGFR	−0.288	< 0.001	−0.202	< 0.001
Nephropathy	0.247	< 0.001	0.304	< 0.001
Kidney failure	0.247	< 0.001	0.233	< 0.001
History of retinal laser treatment	0.283	< 0.001	0.224	< 0.001
History of CVD events	0.202	< 0.001	0.147	< 0.001
Antihypertensive therapy	0.242	< 0.001	0.210	< 0.001
Statin therapy	0.245	< 0.001	0.148	< 0.001

*cfPWV* carotid-femoral pulse wave velocity, *AIx* Augmentation index, *adj-HbA*_*1c*_*-SD* adjusted standard deviation of HbA_1c_, *HbA*_*1c*_*-CV*_*IFCC*_ coefficient of variation of HbA_1c_ based on values in mmol/mol units, *HbA*_*1c*_*-CV*_*NGSP*_ coefficient of variation of HbA_1c_ based on values in % units, *HbA*_*1c*_*-ARV* average real variability of HbA_1c_

History of CVD (cardiovascular disease) events defined as myocardial infarction, coronary revascularization, stroke, lower extremity revascularization or non-traumatic amputation. Kidney failure defined as kidney replacement therapy or eGFR < 15 ml/min/1.73m^2^. Nephropathy defined as severe albuminuria (urinary albumin excretion rate ≥ 300 mg/24 h or ≥ 200 μg/min) or kidney failure

The additional covariates considered in the stepwise variable selection method for multiple linear regression regarding cfPWV were waist-to-height ratio (WHtR), SBP, smoking, LDL-cholesterol, triglycerides, eGFR, kidney failure, history of CVD events, history of retinal laser treatment, antihypertensive therapy (AHT) and statin therapy. In the regression analysis for AIx, the same covariates were considered, with the addition of height, and using MAP instead of SBP. When analysed in separate fully adjusted models (adjusted for sex, age, further variables selected by the stepwise variable selection method, and HbA_1c_-mean), adj-HbA_1c_-SD and HbA_1c_-CV were associated with cfPWV with standardized beta coefficients (st. *β*) of 0.097 (*p* = 0.032) and 0.081 (*p* = 0.046), respectively (Table 3). Similarly, adj-HbA_1c_-SD and HbA_1c_-CV remained significantly associated with AIx (st. *β* 0.070 [*p* = 0.028] and 0.057 [*p* = 0.049], respectively). HbA_1c_-ARV, however, was not associated with cfPWV or AIx in the fully adjusted models, nor did HbA_1c_-mean added as a final adjustment reach statistical significance in any of the models. The adjusted coefficient of determination (*R*^*2*^_*adj*_) of the final models varied between 0.548 and 0.564.

**Table 3:** Measures of arterial stiffness regressed on indices of HbA_1c_ variability in stepwise multivariable linear regression models

ln(cfPWV) (*n* = 335)					AIx (*n* = 653)				
Adjustments	*B*	95% CI	st. *β*	*p* value	Adjustments	*B*	95% CI	st. *β*	*p* value
	ln(adj-HbA_1c_-SD)					ln(adj-HbA_1c_-SD)			
+ Age, Sex	0.136	0.083–0.190	0.208	**< 0.001**	+ Age, Sex	4.256	2.848–5.664	0.186	**< 0.001**
+ SBP	0.118	0.067–0.170	0.180	**< 0.001**	+ MAP	3.146	1.849–4.442	0.138	**< 0.001**
+ Retinopathy	0.089	0.036–0.142	0.136	**0.001**	+ Height	2.771	1.482–4.059	0.121	**< 0.001**
+ AHT	0.077	0.024–0.131	0.118	**0.005**	+ ln(K—eGFR)	2.258	0.951–3.565	0.099	**< 0.001**
+ HbA_1c_-mean	0.064	0.005–0.123	0.097	**0.032**	+ Smoking	1.995	0.684–3.305	0.087	**0.003**
*R*^*2*^_*adj*_	0.562				+ AHT	1.796	0.475–3.117	0.079	**0.008**
					+ HbA_1c_-mean	1.607	0.171–3.042	0.070	**0.028**
					*R*^*2*^_*adj*_	0.551			
	ln(HbA_1c_-CV)					ln(HbA_1c_-CV)			
+ Age, Sex	0.121	0.059–0.182	0.162	** < 0.001**	+ Age, Sex	3.455	1.848–5.061	0.134	**< 0.001**
+ SBP	0.108	0.049–0.166	0.145	**< 0.001**	+ MAP	2.703	1.243–4.163	0.105	**< 0.001**
+ Retinopathy	0.076	0.017–0.135	0.102	**0.012**	+ Height	2.379	0.937–3.822	0.092	**0.001**
+ AHT	0.066	0.007–0.125	0.089	**0.029**	+ ln(K – eGFR)	1.783	0.322–3.243	0.069	**0.017**
+ Statin therapy	0.060	0.001–0.119	0.081	**0.046**	+ Smoking	1.582	0.127–3.037	0.061	**0.033**
+ HbA_1c_-mean	0.060	0.001–0.119	0.081	**0.046**	+ AHT	1.472	0.019–2.925	0.057	**0.047**
*R*^*2*^_*adj*_	0.564				+ HbA_1c_-mean	1.457	0.006–2.908	0.057	**0.049**
					*R*^*2*^_*adj*_	0.550			
	ln(HbA_1c_-ARV)					ln(HbA_1c_-ARV)			
+ Age, Sex	0.109	0.047–0.170	0.145	** < 0.001**	+ Age, Sex	3.241	1.648–4.834	0.125	** < 0.001**
+ SBP	0.094	0.036–0.153	0.126	**0.002**	+ MAP	1.961	0.497–3.425	0.076	**0.009**
+ Retinopathy	0.076	0.019–0.133	0.102	**0.010**	+ Height	1.710	0.268–3.153	0.066	**0.020**
+ AHT	0.066	0.009–0.123	0.088	**0.024**	+ ln(K – eGFR)	1.361	−0.073–2.794	0.053	0.063
+ HbA_1c_-mean	0.047	-0.019–0.112	0.062	**0.161**	+ Smoking	0.976	−0.468–2.420	0.038	0.185
*R*^*2*^_*adj*_	0.558				+ AHT	0.725	−0.731–2.180	0.028	0.329
					+ HbA_1c_-mean	0.194	−1.454–1.842	0.007	**0.817**
					*R*^*2*^_*adj*_	0.548			

Statistically significant *p* values (<0.05) marked in bold. Retinopathy defined as history of retinal laser treatment

*Adj-HbA*_*1c*_*-SD* adjusted standard deviation of HbA_1c_, *HbA*_*1c*_*-CV* coefficient of variation of HbA_1c_, *HbA*_*1c*_*-ARV* average real variability of HbA_1c_, *cfPWV* carotid-femoral pulse wave velocity, *AIx* augmentation index, *SBP* systolic blood pressure, *AHT* antihypertensive therapy, *MAP* mean arterial pressure, *CI* confidence interval for *B*, *st. β* standardized beta coefficient, R^2^_adj_ adjusted coefficient of determination, *K* max(eGFR) + 1

## Discussion

The main finding of this cross-sectional study was an independent association between HbA_1c_ variability (adj-HbA_1c_-SD and HbA_1c_-CV) and arterial stiffness (cfPWV and AIx) in individuals with type 1 diabetes. Notably, these associations were independent of HbA_1c_-mean, which is a consistent finding with other studies evaluating HbA_1c_ variability as a cardiovascular risk factor [29]. Associations observed between ARV, a less established index of GV, and arterial stiffness were diluted to non-significant in multivariable models. HbA_1c_-ARV was also the index of HbA_1c_ variability with highest correlation with HbA_1c_-mean, which may partly explain its poorer survival in models adjusted for HbA_1c_-mean.

To our knowledge, the association between HbA_1c_ variability and arterial stiffness has previously only been studied by Helleputte et al. in a small sample (n = 54) of individuals with type 1 diabetes, in which the individuals studied were more often men (59.3% vs 48.7% in our cfPWV sample), had a shorter duration of diabetes, and were free from known CVD [24]. The authors found no association between HbA_1c_-SD over 10 years and cfPWV. In addition to a smaller sample size, which by itself might explain the lack of association, there were differences to our study related to the exposure and outcome measures. Helleputte et al. used the direct method (with correction by 80%) in the determination of arterial path length for cfPWV measurement, while the subtraction method was used in our study, but had a similar median of cfPWV (8.3 [6.8–10.1] m/s vs 8.5 [7.1–10.8] m/s). Importantly, Helleputte et al. only used one index of HbA_1c_ variability, HbA_1c_-SD, which does not take into account the number, the mean value or the order of the HbA_1c_ measurements, as do adj-HbA_1c_-SD, HbA_1c_-CV and HbA_1c_-ARV, respectively. Also noteworthy, log-transformed indices of HbA_1c_ variability were used in our study to conform them to linear models, while no variable transformations were reported to have been done by Helleputte et al.

The findings of the current study follow our hypothesis raised based on a previous FinnDiane study that showed HbA_1c_ variability to be an independent predictor of incident CVD events [9], and further support this by showing an association with arterial stiffness, which is considered an early marker of CVD and has recently been shown to predict cardiovascular outcomes in individuals with type 1 diabetes [16–18]. Also a recent publication from the CACTI Study pointed towards an association between HbA_1c_-SD and incident CVD events independent of sex, age and type 1 diabetes duration, although this association did not remain statistically significant after adjustments for multiple CVD risk factors [10].

HbA_1c_ values vary spontaneously even in healthy individuals but this variation is considerably lower than that of fasting blood glucose, motivating the use of HbA_1c_ variability as a marker of long-term GV [30]. The association between HbA_1c_ variability and short-term GV is not clear. Although HbA_1c_ level is more influenced by mean blood glucose than by short-term GV, there is a statistically significant association with the latter, too [31]. In a small pilot study using flash glucose monitoring, HbA_1c_ variability was associated with hypoglycaemic indices but not with short-term GV [32]. While no cut-off value for HbA_1c_ variability in type 1 diabetes has been established, an HbA_1c_-CV of approximately 5% has been proposed as a potential threshold for labile HbA_1c_ in type 2 diabetes [30].

A shared consequence of several molecular pathways behind hyperglycaemia-induced vascular damage is increased oxidative stress due to overproduction of superoxides [33]. Interestingly, short-term GV appears to be an even stronger driver of oxidative stress and endothelial dysfunction than sustained hyperglycaemia [34, 35]. These studies were, however, conducted in individuals with type 2 diabetes. Studies on the association between short-term GV and oxidative stress in type 1 diabetes have shown heterogeneous results [36]. Some associations between long-term GV and oxidative stress markers have also been shown [37, 38], but most mechanistic studies have focused on short-term GV, and the true factors behind the ability of HbA_1c_ variability to capture cardiovascular risk are still unknown. The simple interpretation is that of two individuals with similar HbA_1c_-mean values, the one with a more variable HbA_1c_ might spend more time at the extreme ends of the glycaemic range. The sustained long-term effects of a past hyperglycaemic period, entailed in the concept of metabolic memory and possibly mediated through epigenetic changes [39], might require a longer period spent in hyperglycaemia than that captured by short-term GV, which would intriguingly explain the distinct role of long-term GV as a risk factor. On the other hand, HbA_1c_ variability has been associated with a greater risk of severe hypoglycaemic events in individuals with type 2 diabetes [40]. Hypoglycaemia itself is proposed as a cardiovascular risk factor, possibly through hemodynamic changes, arrhythmias, and a combination of oxidative stress, endothelial dysfunction, inflammation and thrombosis, which seem to further increase in response to rebound hyperglycaemia [14, 41–43]. Oxidative stress and endothelial dysfunction are not only suspected to underlie the effects of GV but are also recognized factors in the pathology of arterial stiffness [15], which supports the findings of the present study.

Exogenous insulin administration exposes individuals with type 1 diabetes to marked glycaemic fluctuations. The short-term goal of the current treatment is, by adequate blood glucose monitoring and appropriate insulin administration, to achieve normoglycaemia while minimizing the risk for hypoglycaemia. This is a major challenge as demonstrated by a threefold risk of severe hypoglycaemia in the group receiving intensive treatment in the DCCT trial [44]. Technological advances have been implemented to facilitate a more sophisticated blood glucose control by CGM, continuous subcutaneous insulin infusion (CSII), and even hybrid closed-loop systems combining the two approaches. Indeed, wearing CGM has reduced short-term GV in type 1 diabetes in randomized clinical trials [45, 46]. The benefits with CSII regarding short-term GV have been inconsistent, while an association with lower HbA_1c_ variability has been observed [47]. Pharmaceuticals with the potential to reduce GV include ultra-long-acting insulins as well as oral non-insulin glucose lowering agents, such as glucagon-like peptide-1 (GLP-1) receptor agonists and sodium-glucose co-transporter 2 (SGLT2) inhibitors [30]. However, a change from basal insulin to ultra-long-acting insulin did not have a significant effect on coefficient of variation of HbA_1c_ levels in a study with 90 individuals with type 1 diabetes [48].

The main strengths of this study include the large sample size, the large amount of longitudinal data on HbA_1c_, as well as the use of three different indices of HbA_1c_ variability and two measures of arterial stiffness, cfPWV being the gold standard measure. The study population has gone through a comprehensive characterization allowing for a wide range of adjustments in the analyses. We acknowledge several limitations in this study. The observational cross-sectional study design only allows us to speculate about causality. There is, however, a retrospective longitudinal dimension to the exposure, as the indices of HbA_1c_ variability were calculated from serial measurements. Due to the retrospective observational design, the assessment intervals of HbA_1c_ were not predefined, although individuals with type 1 diabetes often do attend regular check-ups including the assessment of HbA_1c_. To counterbalance this issue, we required a minimum of five measurements per individual, as opposed to three measurements, which is the minimum for calculating SD. A possible source of bias is that the methods for HbA_1c_ measurement have changed over time and may differ between laboratories. This is, however, more likely to affect the inter-individual than the intra-individual variability, which was assessed in this study. Also, the nationwide quality surveys of HbA_1c_ measurements in Finland have shown a high correlation with the DCCT reference method [49]. Although a large set of relevant clinical covariates were included in the analysis, the *R*^*2*^_*adj*_ of the regression models was rather low, indicating the possibility of residual confounding. With the available data, we cannot conclude, to what extent the observed associations could be attributed to hypoglycaemia instead of long-term GV per se. In terms of statistical power, we acknowledge that despite a large study population in the category of cohorts of individuals with type 1 diabetes, the sample size was relatively small for cfPWV. This would, however, rather dilute the finding than cause false positive findings. Lastly, the external validity of the results is limited due to including individuals from only one study centre, as this was the site for arterial stiffness assessment.

## Conclusions

In this study, we observed a novel association between HbA_1c_ variability and arterial stiffness in individuals with type 1 diabetes, independent of HbA_1c_-mean and other relevant clinical covariates. As neither long-term GV nor arterial stiffness has yet been implemented in regular clinical assessment, further observational and interventional studies on the association might offer new targets for the early prevention of cardiovascular complications of diabetes. In practice, the findings indicate a potential value in including indices of HbA_1c_ variability in the cardiovascular risk assessment of individuals with type 1 diabetes. Further studies are also needed to establish definitions and clinical thresholds for indices of HbA_1c_ variability, as well as to confirm whether targeting long-term glycaemic variability using new technology and add-on medications, such as GLP-1 receptor agonists and SGLT2 inhibitors, will reduce the cardiovascular risk for individuals with type 1 diabetes.

## Data Availability

Individual-level data for the study participants are not publicly available because of the restrictions due to the study consent provided by the participant at the time of data collection.

## Abbreviations

adj-HbA_1c_-SD: Adjusted standard deviation of HbA_1c_
AHT: Antihypertensive therapy
AIx: Augmentation index
cfPWV: Carotid-femoral pulse wave velocity
CGM: Continuous glucose monitoring
CSII: Continuous subcutaneous insulin infusion
FinnDiane: The Finnish Diabetic Nephropathy Study
GV: Glycaemic variability
HbA_1c_-ARV: Average real variability of HbA_1c_
HbA_1c_-CV: Coefficient of variation of HbA_1c_
MAP: Mean arterial pressure
*r*_*s*_: Spearman’s rank correlation coefficient
*R*^*2*^_*adj*_: Adjusted coefficient of determination
SBP: Systolic blood pressure
SGLT2: Sodium-glucose co-transporter 2
st. *β*: Standardized beta coefficient

## References

[1] Harjutsalo V, Pongrac Barlovic D, Groop PH (2021). Long-term population-based trends in the incidence of cardiovascular disease in individuals with type 1 diabetes from Finland: a retrospective, nationwide, cohort study. Lancet Diabetes Endocrinol.

[2] Livingstone SJ, Levin D, Looker HC, Lindsay RS, Wild SH, Joss N (2015). Estimated life expectancy in a Scottish cohort with type 1 diabetes, 2008–2010. JAMA.

[3] Groop PH, Thomas M, Feodoroff M, Forsblom C, Harjutsalo V, FinnDiane Study Group (2018). Excess mortality in patients with type 1 diabetes without albuminuria-separating the contribution of early and late risks. Diabetes Care.

[4] Nathan DM, Cleary PA, Backlund JYC, Genuth SM, Lachin JM, Orchard TJ (2005). Intensive diabetes treatment and cardiovascular disease in patients with type 1 diabetes. N Engl J Med.

[5] Lind M, Svensson AM, Kosiborod M, Gudbjörnsdottir S, Pivodic A, Wedel H (2014). Glycemic control and excess mortality in type 1 diabetes. N Engl J Med.

[6] Gerstein HC, Miller ME, Byington RP, Goff DC, Bigger JT, Action to Control Cardiovascular Risk in Diabetes Study Group (2008). Effects of intensive glucose lowering in type 2 diabetes. N Engl J Med.

[7] Huang D, Huang YQ, Zhang QY, Cui Y, Mu TY, Huang Y (2021). Association between long-term visit-to-visit hemoglobin A1c and cardiovascular risk in type 2 diabetes: the ACCORD trial. Front Cardiovasc Med.

[8] Battelino T, Danne T, Bergenstal RM, Amiel SA, Beck R, Biester T (2019). Clinical targets for continuous glucose monitoring data interpretation: recommendations from the international consensus on time in range. Diabetes Care.

[9] Wadén J, Forsblom C, Thorn LM, Gordin D, Saraheimo M, Groop PH (2009). A1C variability predicts incident cardiovascular events, microalbuminuria, and overt diabetic nephropathy in patients with type 1 diabetes. Diabetes.

[10] Horton WB, Snell-Bergeon JK (2023). HbA1c variability metrics predict coronary artery calcium and cardiovascular events in type 1 diabetes: the CACTI study. J Clin Endocrinol Metab.

[11] Walker GS, Cunningham SG, Sainsbury CAR, Jones GC (2017). HbA1c variability is associated with increased mortality and earlier hospital admission in people with Type 1 diabetes. Diabet Med J Br Diabet Assoc.

[12] Rotbain Curovic V, Theilade S, Winther SA, Tofte N, Tarnow L, Jorsal A (2021). Visit-to-visit variability of clinical risk markers in relation to long-term complications in type 1 diabetes. Diabet Med J Br Diabet Assoc.

[13] Steineck I, Cederholm J, Eliasson B, Rawshani A, Eeg-Olofsson K, Svensson AM (2015). Insulin pump therapy, multiple daily injections, and cardiovascular mortality in 18,168 people with type 1 diabetes: observational study. BMJ.

[14] Papachristoforou E, Lambadiari V, Maratou E, Makrilakis K (2020). Association of glycemic indices (Hyperglycemia, glucose variability, and hypoglycemia) with oxidative stress and diabetic complications. J Diabetes Res.

[15] Vatner SF, Zhang J, Vyzas C, Mishra K, Graham RM, Vatner DE (2021). vascular stiffness in aging and disease. Front Physiol.

[16] Tynjälä A, Forsblom C, Harjutsalo V, Groop PH, Gordin D, FinnDiane Study Group (2020). Arterial stiffness predicts mortality in individuals with type 1 diabetes. Diabetes Care.

[17] Tougaard NH, Theilade S, Winther SA, Tofte N, Ahluwalia TS, Hansen TW (2020). Carotid-femoral pulse wave velocity as a risk marker for development of complications in type 1 diabetes mellitus. J Am Heart Assoc.

[18] Tynjälä A, Forsblom C, Harjutsalo V, Groop PH, Gordin D (2021). Response to comment on comment on Tynjälä et al. Arterial Stiffness predicts mortality in individuals with type 1 diabetes 2020;43:2266-2271. Diabetes Care.

[19] Wakasugi S, Mita T, Katakami N, Okada Y, Yoshii H, Osonoi T (2021). Associations between continuous glucose monitoring-derived metrics and arterial stiffness in Japanese patients with type 2 diabetes. Cardiovasc Diabetol.

[20] Foreman YD, van Doorn WPTM, Schaper NC, van Greevenbroek MMJ, van der Kallen CJH, Henry RMA (2021). Greater daily glucose variability and lower time in range assessed with continuous glucose monitoring are associated with greater aortic stiffness: the Maastricht Study. Diabetologia.

[21] Zhang Y, Wu S, Li M, Wang T, Xu M, Lu J (2021). Long-term glycemic variability is associated with arterial stiffness in Chinese adults. Front Endocrinol.

[22] Gordin D, Rönnback M, Forsblom C, Mäkinen V, Saraheimo M, Groop PH (2008). Glucose variability, blood pressure and arterial stiffness in type 1 diabetes. Diabetes Res Clin Pract.

[23] Cesana F, Giannattasio C, Nava S, Soriano F, Brambilla G, Baroni M (2013). Impact of blood glucose variability on carotid artery intima media thickness and distensibility in type 1 diabetes mellitus. Blood Press.

[24] Helleputte S, Calders P, Rodenbach A, Marlier J, Verroken C, De Backer T (2022). Time-varying parameters of glycemic control and glycation in relation to arterial stiffness in patients with type 1 diabetes. Cardiovasc Diabetol.

[25] Laurent S, Cockcroft J, Van Bortel L, Boutouyrie P, Giannattasio C, Hayoz D (2006). Expert consensus document on arterial stiffness: methodological issues and clinical applications. Eur Heart J.

[26] Mena L, Pintos S, Queipo NV, Aizpúrua JA, Maestre G, Sulbarán T (2005). A reliable index for the prognostic significance of blood pressure variability. J Hypertens.

[27] Wilkinson IB, Fuchs SA, Jansen IM, Spratt JC, Murray GD, Cockcroft JR (1998). Reproducibility of pulse wave velocity and augmentation index measured by pulse wave analysis. J Hypertens.

[28] Van Bortel LM, Laurent S, Boutouyrie P, Chowienczyk P, Cruickshank JK, De Backer T (2012). Expert consensus document on the measurement of aortic stiffness in daily practice using carotid-femoral pulse wave velocity. J Hypertens.

[29] Gorst C, Kwok CS, Aslam S, Buchan I, Kontopantelis E, Myint PK (2015). Long-term glycemic variability and risk of adverse outcomes: a systematic review and meta-analysis. Diabetes Care.

[30] Monnier LO, Owens D, Colette C, Bonnet F (2021). Glycaemic variabilities: key questions in pursuit of clarity. Diabetes Metab.

[31] McCarter RJ, Hempe JM, Chalew SA (2006). Mean blood glucose and biological variation have greater influence on HbA1c levels than glucose instability: an analysis of data from the diabetes control and complications trial. Diabetes Care.

[32] Tokutsu A, Okada Y, Torimoto K, Tanaka Y (2021). Relationship between glycemic intraday variations evaluated in continuous glucose monitoring and HbA1c variability in type 2 diabetes: pilot study. Diabetol Metab Syndr.

[33] Brownlee M (2001). Biochemistry and molecular cell biology of diabetic complications. Nature.

[34] Monnier L, Mas E, Ginet C, Michel F, Villon L, Cristol JP (2006). Activation of oxidative stress by acute glucose fluctuations compared with sustained chronic hyperglycemia in patients with type 2 diabetes. JAMA.

[35] Ceriello A, Esposito K, Piconi L, Ihnat MA, Thorpe JE, Testa R (2008). Oscillating glucose is more deleterious to endothelial function and oxidative stress than mean glucose in normal and type 2 diabetic patients. Diabetes.

[36] Valente T, Valente F, Lucchesi MBB, Punaro GR, Mouro MG, Gabbay MAL (2021). Relationship between short and long-term glycemic variability and oxidative stress in type 1 diabetes mellitus under daily life insulin treatment. Arch Endocrinol Metab.

[37] Chang CM, Hsieh CJ, Huang JC, Huang IC (2012). Acute and chronic fluctuations in blood glucose levels can increase oxidative stress in type 2 diabetes mellitus. Acta Diabetol.

[38] Rodrigues R, de Medeiros LA, Cunha LM, da Garrote-Filho MS, Bernardino Neto M, Jorge PT (2018). Correlations of the glycemic variability with oxidative stress and erythrocytes membrane stability in patients with type 1 diabetes under intensive treatment. Diabetes Res Clin Pract..

[39] Reddy MA, Zhang E, Natarajan R (2015). Epigenetic mechanisms in diabetic complications and metabolic memory. Diabetologia.

[40] Long C, Tang Y, Huang J, Liu S, Xing Z (2022). Association of long-term visit-to-visit variability of HbA1c and fasting glycemia with hypoglycemia in type 2 diabetes mellitus. Front Endocrinol.

[41] International Hypoglycaemia Study Group (2019). Hypoglycaemia, cardiovascular disease, and mortality in diabetes: epidemiology, pathogenesis, and management. Lancet Diabetes Endocrinol.

[42] Ceriello A, Novials A, Ortega E, La Sala L, Pujadas G, Testa R (2012). Evidence that hyperglycemia after recovery from hypoglycemia worsens endothelial function and increases oxidative stress and inflammation in healthy control subjects and subjects with type 1 diabetes. Diabetes.

[43] Ceriello A, Novials A, Ortega E, Pujadas G, La Sala L, Testa R (2014). Hyperglycemia following recovery from hypoglycemia worsens endothelial damage and thrombosis activation in type 1 diabetes and in healthy controls. Nutr Metab Cardiovasc Dis NMCD.

[44] Hypoglycemia in the Diabetes Control and Complications Trial (1997). The diabetes control and complications trial research group. Diabetes.

[45] Beck RW, Riddlesworth T, Ruedy K, Ahmann A, Bergenstal R, Haller S (2017). Effect of continuous glucose monitoring on glycemic control in adults with type 1 diabetes using insulin injections: the diamond randomized clinical trial. JAMA.

[46] Lind M, Polonsky W, Hirsch IB, Heise T, Bolinder J, Dahlqvist S (2017). Continuous glucose monitoring vs conventional therapy for glycemic control in adults with type 1 diabetes treated with multiple daily insulin injections: the gold randomized clinical trial. JAMA.

[47] Scott ES, McGrath RT, Januszewski AS, Calandro D, Hardikar AA, O’Neal DN (2019). HbA1c variability in adults with type 1 diabetes on continuous subcutaneous insulin infusion (CSII) therapy compared to multiple daily injection (MDI) treatment. BMJ Open.

[48] Watanabe H, Takahara M, Katakami N, Shimomura I (2022). Changes of HbA1c variability after the switch to a longer-acting insulin analog in people with type 1 diabetes. J Diabetes Investig.

[49] Penttilä IM, Halonen T, Punnonen K, Tiikkainen U (2005). Best use of the recommended IFCC reference method, material and values in HbA1C analyses. Scand J Clin Lab Invest.

