# Correction: Higher HbA_1c_ variability is associated with increased arterial stiffness in individuals with type 1 diabetes

**DOI:** 10.1186/s12933-023-01813-8

**Published:** 2023-04-08

**Authors:** Anniina Tynjälä, Valma Harjutsalo, Fanny Jansson Sigfrids, Per-Henrik Groop, Daniel Gordin, the FinnDiane Study Group

**Affiliations:** 1 Folkhälsan Institute of Genetics, Folkhälsan Research Center, Helsinki, Finland; 2 Department of Nephrology, University of Helsinki and Helsinki University Hospital, Helsinki, Finland; 3 Research Program for Clinical and Molecular Metabolism, Faculty of Medicine, University of Helsinki, Helsinki, Finland; 4 Minerva Foundation Institute for Medical Research, Helsinki, Finland; 5 Department of Diabetes, Central Clinical School, Monash University, Melbourne, Australia; 6 Joslin Diabetes Center, Harvard Medical School, Boston, MA USA

**Correction** **:** **Cardiovascular Diabetology (2023) 22:47** **https://doi.org/10.1186/s12933-023-01770-2**

Following publication of the original article [1], the authors noticed the errors occurred in Table 3. During proof-correction stage, the typesetter has inadvertently given bold for p-values. These values have now been corrected with this erratum.

**Table 3:** Measures of arterial stiffness regressed on indices of HbA_1c_ variability in stepwise multivariable linear regression models

ln(cfPWV) (*n* = 335)					AIx (*n* = 653)				
Adjustments	*B*	95% CI	st. *β*	*p* value	Adjustments	*B*	95% CI	st. *β*	*p* value
	ln(**adj-HbA**_**1c**_**-SD**)					ln(**adj-HbA**_**1c**_**-SD**)			
+ Age, Sex	0.136	0.083–0.190	0.208	** < 0.001**	+ Age, Sex	4.256	2.848–5.664	0.186	** < 0.001**
+ SBP	0.118	0.067–0.170	0.180	** < 0.001**	+ MAP	3.146	1.849–4.442	0.138	** < 0.001**
+ Retinopathy	0.089	0.036–0.142	0.136	**0.001**	+ Height	2.771	1.482–4.059	0.121	** < 0.001**
+ AHT	0.077	0.024–0.131	0.118	**0.005**	+ ln(K – eGFR)	2.258	0.951–3.565	0.099	** < 0.001**
+ HbA_1c_-mean	0.064	0.005–0.123	0.097	**0.032**	+ Smoking	1.995	0.684–3.305	0.087	**0.003**
*R*^*2*^_*adj*_	0.562				+ AHT	1.796	0.475–3.117	0.079	**0.008**
					+ HbA_1c_-mean	1.607	0.171–3.042	0.070	**0.028**
					*R*^*2*^_*adj*_	0.551			
	ln(**HbA**_**1c**_**-CV**)					ln(**HbA**_**1c**_**-CV**)			
+ Age, Sex	0.121	0.059–0.182	0.162	** < 0.001**	+ Age, Sex	3.455	1.848–5.061	0.134	** < 0.001**
+ SBP	0.108	0.049–0.166	0.145	** < 0.001**	+ MAP	2.703	1.243–4.163	0.105	** < 0.001**
+ Retinopathy	0.076	0.017–0.135	0.102	**0.012**	+ Height	2.379	0.937–3.822	0.092	**0.001**
+ AHT	0.066	0.007–0.125	0.089	**0.029**	+ ln(K – eGFR)	1.783	0.322–3.243	0.069	**0.017**
+ Statin therapy	0.060	0.001–0.119	0.081	**0.046**					
+ HbA_1c_-mean	0.060	0.001–0.119	0.081	**0.046**	+ Smoking	1.582	0.127–3.037	0.061	**0.033**
*R*^*2*^_*adj*_	0.564				+ AHT	1.472	0.019–2.925	0.057	**0.047**
					+ HbA_1c_-mean	1.457	0.006–2.908	0.057	**0.049**
					*R*^*2*^_*adj*_	0.550			
	ln(**HbA**_**1c**_**-ARV**)					ln(**HbA**_**1c**_**-ARV**)			
+ Age, Sex	0.109	0.047–0.170	0.145	** < 0.001**	+ Age, Sex	3.241	1.648–4.834	0.125	** < 0.001**
+ SBP	0.094	0.036–0.153	0.126	**0.002**	+ MAP	1.961	0.497–3.425	0.076	**0.009**
+ Retinopathy	0.076	0.019–0.133	0.102	**0.010**	+ Height	1.710	0.268–3.153	0.066	**0.020**
+ AHT	0.066	0.009–0.123	0.088	**0.024**	+ ln(K – eGFR)	1.361	-0.073–2.794	0.053	0.063
+ HbA_1c_-mean	0.047	-0.019–0.112	0.062	0.161	+ Smoking	0.976	-0.468–2.420	0.038	0.185
*R*^*2*^_*adj*_	0.558				+ AHT	0.725	-0.731–2.180	0.028	0.329
					+ HbA_1c_-mean	0.194	-1.454–1.842	0.007	0.817
					*R*^*2*^_*adj*_	0.548			

Adj-HbA_1c_-SD: adjusted standard deviation of HbA_1c_, HbA_1c_-CV: coefficient of variation of HbA_1c_, HbA_1c_-ARV: average real variability of HbA_1c_, cfPWV: carotid-femoral pulse wave velocity, AIx: augmentation index, SBP: systolic blood pressure, AHT: antihypertensive therapy, MAP: mean arterial pressure, CI: confidence interval for *B*, st. *β*: standardized beta coefficient, *R*^*2*^_*adj*_*:* adjusted coefficient of determination, K: max(eGFR) + 1. Retinopathy defined as history of retinal laser treatment

The original article has been corrected.

## References

[1] Tynjälä A, Harjutsalo V, Sigfrids FJ, Groop P-H, Gordin D, on behalf of the FinnDiane Study Group (2023). Higher HbA_1c_ variability is associated with increased arterial stiffness in individuals with type 1 diabetes. Cardiovasc Diabetol.

